# A New Method for Absolute Pose Estimation with Unknown Focal Length and Radial Distortion

**DOI:** 10.3390/s22051841

**Published:** 2022-02-25

**Authors:** Kai Guo, Hu Ye, Honglin Chen, Xin Gao

**Affiliations:** Northwest Institute of Nuclear Technology, Xi’an 710024, China; yxc0228@163.com (H.Y.); chenhhll15@163.com (H.C.); glonor@163.com (X.G.)

**Keywords:** radial distortion, absolute pose, unknown focal length, multiple radial distortion models, single solution

## Abstract

Estimating the absolute pose of a camera is one of the key steps for computer vision. In some cases, especially when using a wide-angle or zoom lens, the focal length and radial distortion also need to be considered. Therefore, in this paper, an efficient and robust method for a single solution is proposed to estimate the absolute pose for a camera with unknown focal length and radial distortion, using three 2D–3D point correspondences and known camera position. The problem is decomposed into two sub-problems, which makes the estimation simpler and more efficient. The first sub-problem is to estimate the focal length and radial distortion. An important geometric characteristic of radial distortion, that the orientation of the 2D image point with respect to the center of distortion (i.e., principal point in this paper) under radial distortion is unchanged, is used to solve this sub-problem. The focal length and up to four-order radial distortion can be determined with this geometric characteristic, and it can be applied to multiple distortion models. The values with no radial distortion are used as the initial values, which are close to the global optimal solutions. Then, the sub-problem can be efficiently and accurately solved with the initial values. The second sub-problem is to determine the absolute pose with geometric linear constraints. After estimating the focal length and radial distortion, the undistorted image can be obtained, and then the absolute pose can be efficiently determined from the point correspondences and known camera position using the undistorted image. Experimental results indicate this method’s accuracy and numerical stability for pose estimation with unknown focal length and radial distortion in synthetic data and real images.

## 1. Introduction

Retrieving the absolute pose of a camera from *n* 2D–3D point correspondences is one of the key steps in computer vision and SfM (structure from motion) [1,2,3,4,5,6]. Many approaches have been proposed to solve this problem, which are named PnP solvers [7,8,9,10,11,12,13] when the intrinsic camera parameters are all known as prior knowledge. The difference in the number of point correspondences makes both the ideas and the number of estimated parameters of PnP solvers different. When there is no prior knowledge except the intrinsic camera parameters, three 2D–3D point correspondences is the minimal subset, and these are called P3P solvers [14,15,16] and they can solve all six degrees of freedom of the camera pose. In practical applications, some intrinsic camera parameters may be unknown, and accordingly, many methods are proposed to work with these cases. When the focal length is unknown, a minimum of four 2D–3D point correspondences is required to estimate the absolute pose, and these corresponding methods are called P4Pf solvers [17]. Theoretically, one 2D–3D point correspondence can give two constraints, and hence eight unknown parameters can be estimated with four 2D–3D point correspondences. This means that another radial distortion parameter can be determined when the focal length is solved. These corresponding methods are called P4Pfr solvers [18,19,20,21]. If there are five 2D–3D point correspondences, up to ten unknown parameters can be estimated. Hence, we can solve three more unknown parameters besides the camera pose and focal length. The three other unknown parameters may be three radial distortion coefficients, and the corresponding methods are called P5Pfr solvers [9,17]. The three unknown parameters may alternatively be the radial distortion coefficient and two parameters of the principal point. The corresponding methods are called P5Pfrp solvers [17]. If there are at least six 2D–3D point correspondences, all the intrinsic and extrinsic camera parameters can be estimated linearly, which is known as Direct Linear Transform (DLT) [17,22].

All the above methods work in cases wherein the six camera pose parameters are all unknown. When some camera pose parameters are known in advance, which allows us to work with fewer degrees of extrinsic camera parameters, the problem can be simplified, and more unknown parameters can be estimated with the same number of 2D–3D point correspondences compared to the methods mentioned above. With the development of technology, position and orientation devices are becoming cheaper, smaller and more accurate, such as in the case of RTK and IMUs [12,23,24,25]. Hence, mounting these devices on cameras is becoming increasingly popular in real scenarios. When the vertical direction of the camera is measured by IMUs, two orientation parameters can be obtained, and many corresponding methods are then proposed to estimate the camera pose or intrinsic camera parameters using knowledge of the vertical direction [26,27,28].

The existing methods can be divided into two categories for camera pose estimation. The first category estimates the relative pose from multiple views or two cameras [6,29,30,31,32,33,34]. The second category estimates the absolute pose from a single image [5,15,18,19,20,35]. In this paper, we focus on the latter. To the best of our knowledge, most existing methods just use the orientation parameters of the camera pose, taken as prior knowledge [12,23,24,25,26,27,28], and few methods use the position parameters as prior knowledge [36,37], which has prompted us to use the camera position for pose and partial intrinsic parameter estimation in this paper. Hence, to estimate the camera pose, this paper focuses on a case wherein the camera position is known. In addition, some of the above methods use an ideal pinhole camera without distortion, and so we now need to estimate some intrinsic parameters, i.e., focal length and radial distortion. This scenario usually arises when using a zoom lens with heavy distortion. In practical applications, the focal length is often unknown (e.g., zoom lens or fisheye lens). In missile range testing, for example, attitude measurement based on fixed cameras with zoom and short focal lenses is an important test [37], and hence the radial distortion, focal length and pose need to be estimated. In addition, with the increasing prominence of the social security problem, visual monitoring cameras (VMC) are used widely. In general, the position of the VMC is fixed, and the lens orientation can be changed. In these cases, the focal length can be changed online, and a large field of view is required, which leads to heavy radial distortion. If heavy distortion occurs, the camera’s pose cannot be estimated directly because the 2D–3D point correspondences are invalid. In this case, radial distortion must be taken into account [38,39]. There are many radial distortion models, such as the traditional model, the division model, and others [40,41,42,43]. The traditional model was first proposed by Brown in 1971 [44], and the division model was first proposed by Fitzgibbon in 2001 [34]. In the existing literature, the traditional model [45,46,47,48] and division model [49,50] are widely used, and the division model is the most popular because it can result in simpler equation systems [5]. Depending on the number of parameters, the radial distortion models can be divided into one-parameter models [6,18,29,51,52,53], two-parameter models [22,54], three-parameter models [35] and arbitrary parameter models [5,55]. According to the existing literature, it has been demonstrated that distortion is mainly dominated by the first two items [41]. Hence, most of the existing methods use a one-parameter model or a two-parameter model, as is the case in this paper.

In this paper, three 2D–3D point correspondences are used to estimate the absolute pose when the focal length and radial distortion of the camera are unknown. Since six constraints can be given by three correspondences, this is the minimal subset for the case. The problem in this paper is decomposed into two sub-problems, which makes the estimation simpler and more efficient. The first sub-problem is to estimate the focal length and radial distortion. An important geometric characteristic of radial distortion, that the orientation of the image point with respect to the center of distortion (i.e., the principal point in this paper) under conditions of radial distortion is unchanged, is used to solve this sub-problem. The focal length and up to four-order radial distortion can be determined iteratively with this geometric characteristic, and can work with multiple distortion models, such as the division model and the traditional model (Brown model). The values estimated without considering the radial distortion can be used as the initial values for iteration, and these are close to the global optimal solutions. Consequently, the sub-problem can be efficiently and accurately solved with these initial values. The second sub-problem is to determine the camera pose with geometric linear constraints after estimating the focal length and radial distortion. Since undistorted images can then be given, we can obtain valid 2D–3D point correspondences, and then camera pose estimation becomes simple.

The proposed method can be used for cases wherein a zoom lens or fisheye lens is used, and the imaging position is set at a distance from the center of the image. The experimental results indicate that our proposed method has higher accuracy and better numerical stability for pose estimation from synthetic data and real images when focal length and radial distortion are unknown.

This paper is organized as follows. Section 2 presents the new method for camera pose estimation when focal length and radial distortion are unknown. Section 3 presents the results on numerical stability and noise sensitivity in the synthetic data and real images. Section 4 presents the discussion. Section 5 presents conclusions.

## 2. Problem and Method Statement

### 2.1. Problem Statement

**Figure 1:**
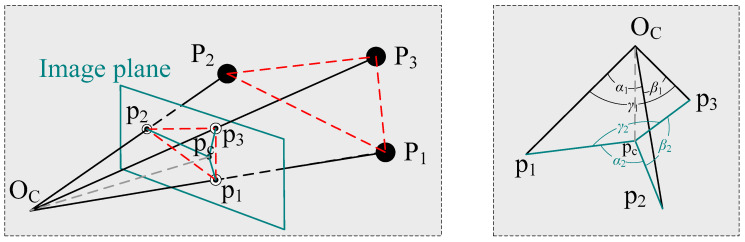
The geometric construction of our problem without radial distortion. The red dashed lines are the distances and their projections between each sets of two 3D points; the gray dashed line is the focal length.

A standard pinhole camera model is used in this paper. This paper uses three 2D–3D point correspondences and the known camera position to estimate the position with an unknown focal length and radial distortion. Up to four-order radial distortion can be estimated efficiently, and our proposed method can work with both the division distortion model and the traditional distortion model. The geometric construction of our problem without radial distortion is illustrated in Figure 1.

In Figure 1, *P_i_* (*i* = 1, 2, 3) is the known 3D control point and *p_i_* is its 2D image projection without radial distortion. *p_c_* is the principal point, which is the center of the image. *O_C_* is the known camera position. Since the radial distortion exists, we obtain only the distorted 2D image point $p_{i}^{d}\;\left(u_{i}^{d}\;v_{i}^{d}\right)$, and the undistorted 2D image point *p_i_* is unknown in real scenarios. In this paper, our core work is to estimate the camera pose when the focal length and radial distortion are unknown from the 3D control points *P_i_* and the distorted 2D image points $p_{i}^{d}$.

### 2.2. Radial Distortion and Focal Length Estimation

Many distortion models have been proposed in the existing literature, and for most digital cameras, the main distortion is radial distortion. Two models are usually used for radial distortion, which are respectively the division model and the traditional model. The division model is written as

(1) $$p_{i}=\frac{p_{i}^{d}}{1+k_{1}r_{i}^{2}+k_{2}r_{i}^{4}+k_{3}r_{i}^{6}+\cdots }$$

The traditional model is written as

(2) $$p_{i}=p_{i}^{d}\left(1+k_{1}r_{i}^{2}+k_{2}r_{i}^{4}+k_{3}r_{i}^{6}+\cdots \right)$$

**Figure 2:**
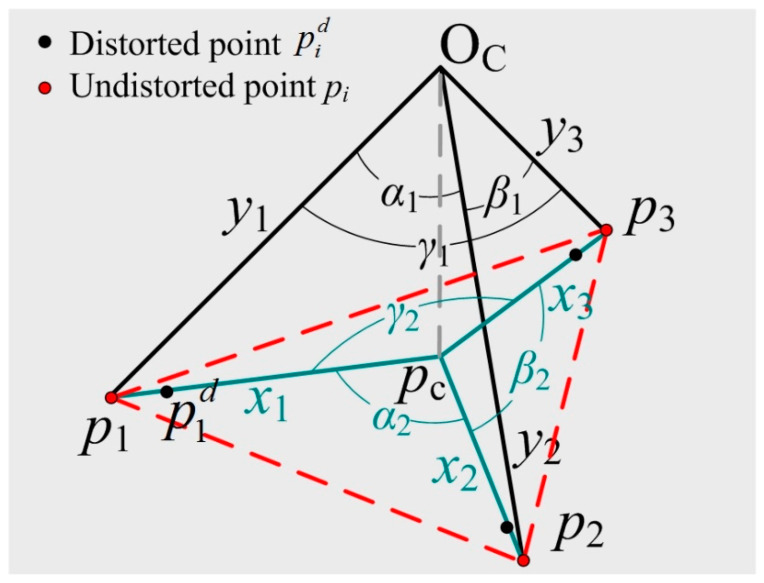
Details of our problem with radial distortion. The green solid line is the distance between the undistorted point *p_i_* and the center of distortion *p_c_*, which is denoted as *x_i_*. The black solid line is the distance between the undistorted point *p_i_* and the camera position *O_C_*, which is denoted as *y_i_*.

Here, *k_i_* is the radial distortion coefficient and *r_i_* = ‖$p_{i}^{d}$‖ is the distance between the distorted 2D point $p_{i}^{d}$ and the center of distortion. The radial distortion is mainly dominated by the first two items [41], and we hence only consider the two items in this paper. It can be seen that no matter which model is used, the orientation of the image point with respect to the center of distortion (i.e., the principal point in this paper) under radial distortion is unchanged, and only the distance changes. This important and key geometric characteristic of radial distortion has encouraged us to propose a new method to estimate the focal length and radial distortion. According to these characteristics, the detailed geometric construction of our problem with radial distortion is illustrated in Figure 2.

In Figure 2, $∠p_{1}O_{c}p_{2}$, $∠p_{2}O_{c}p_{3}$, $∠p_{3}O_{c}p_{1}$, are denoted as *α*_1_, *β*_1_, *γ*_1_ respectively, and can be computed with triangles $△P_{1}O_{c}P_{2}$, $△P_{2}O_{c}P_{3}$, $△P_{3}O_{c}P_{1}$, as shown in Figure 1. $∠p_{1}p_{c}p_{2}$, $∠p_{2}p_{c}p_{3}$, $∠p_{3}p_{c}p_{1}$ are denoted as *α*_2_, *β*_2_, *γ*_2_, respectively. Here, the principal point *p_c_* and the distorted imaging points $p_{i}^{d}$ are known, but the undistorted imaging points *p_i_* are unknown. Note that the orientation of the distorted image point with respect to the center of the distortion is unchanged under the radial distortion, and therefore *α*_2_, *β*_2_, *γ*_2_ can be computed with $△p_{1}^{d}p_{c}p_{2}^{d}$, $△p_{2}^{d}p_{c}p_{3}^{d}$, $△p_{3}^{d}p_{c}p_{1}^{d}$, respectively. In the next step of the derivation using our method, only the distances between the undistorted image points and the center of distortion are used. These are unknown and need to be computed. Therefore, the derivation does not involve the distortion coefficients and does not solve the distortion coefficients directly.

Let *p_c_p_i_ = x_i_* and *O_c_p_i_ = y_i_*, and since △*p*_1_*p_c_p*_2_ and △*p*_1_*O_c_p*_2_ have the common edge *p*_1_*p*_2_, an equation by the cosine law can be given

(3) $$y_{1}^{2}+y_{2}^{2}-2y_{1}y_{2}\cos\alpha_{1}=x_{1}^{2}+x_{2}^{2}-2x_{1}x_{2}\cos\alpha_{2}$$

In Figure 2, *O_c_p_c_ = f* is the focal length, which is perpendicular to the plane *p*_1_*p*_2_*p*_3_. Hence, according to the triangle △*O_c_p_c_p_i_*, an equation can be given by the Pythagorean Theorem

(4) $$f^{2}+x_{i}^{2}=y_{i}^{2}$$

Take Equation (4) into Equation (3), and rewrite it as

$$f^{2}+x_{1}x_{2}\cos\alpha_{2}=\sqrt{x_{1}^{2}+f^{2}}\sqrt{x_{2}^{2}+f^{2}}\cos\alpha_{1}$$

Similarly, the other two equations can be given

(5) $$\left\{ \begin{array}{l} f^{2}+x_{2}x_{3}\cos\beta_{2}=\sqrt{x_{2}^{2}+f^{2}}\sqrt{x_{3}^{2}+f^{2}}\cos\beta_{1}\\ f^{2}+x_{3}x_{1}\cos\gamma_{2}=\sqrt{x_{3}^{2}+f^{2}}\sqrt{x_{1}^{2}+f^{2}}\cos\gamma_{1} \end{array}\right.$$

We set $\frac{x_{i}}{f}$ to be equal to *f_i_*, and then a system of equations with three variables was given, as follows.

(6) $$\left\{ \begin{array}{l} 1+f_{1}f_{2}\cos\alpha_{2}=\sqrt{1+f_{1}^{2}}\sqrt{1+f_{2}^{2}}\cos\alpha_{1}\\ 1+f_{2}f_{3}\cos\beta_{2}=\sqrt{1+f_{2}^{2}}\sqrt{1+f_{3}^{2}}\cos\beta_{1}\\ 1+f_{3}f_{1}\cos\gamma_{2}=\sqrt{1+f_{3}^{2}}\sqrt{1+f_{1}^{2}}\cos\gamma_{1} \end{array}\right.$$

The Levenberg–Marquardt (LM) algorithm [50] can be used to solve this system. It is an iterative solver, and good initial solutions to *f_i_* are needed to obtain the global optimal solutions. Choosing the initial solutions is one of the key steps in this paper. Here, when we choose the initial solutions, the radial distortion is not considered. Then, the initial solutions can be given by some existing methods [15,16]. The initial solutions without radial distortion are used for Equation (6), and the *f_i_* can be computed iteratively. The proposed method with these initial solutions can converge to the global optimal solution, which will be shown in Section 3.

After obtaining the value of *f_i_*, the focal length and radial distortion coefficients can be computed with different radial distortion models respectively, i.e., the division model and traditional model.

(1) The division model. This two-parameter model is given by the formula

(7) $$p_{i}=\frac{p_{i}^{d}}{1+k_{1}r_{i}^{2}+k_{2}r_{i}^{4}}$$

Here, *k_j_* (*j* = 1, 2) is the radial distortion coefficient and $r_{i}=x_{i}^{d}=\left\|p_{i}^{d}\right\|$ is the distance between the distorted point $p_{i}^{d}$ and the principal point. Then we can obtain

(8) $$x_{i}=\frac{x_{i}^{d}}{1+k_{1}{\left(x_{i}^{d}\right)}^{2}+k_{2}{\left(x_{i}^{d}\right)}^{4}}$$

$x_{i}^{d}=r_{i}=\sqrt{{\left(u_{i}^{d}\right)}^{2}+{\left(v_{i}^{d}\right)}^{2}}$ is known, and a system of polynomial equations can be obtained with the *f_i_* computed by the LM algorithm

(9) $$\left\{ \begin{array}{l} f_{1}f+f_{1}r_{1}^{2}\cdot k_{1}f+f_{1}r_{1}^{4}\cdot k_{2}f=r_{1}\\ f_{2}f+f_{2}r_{2}^{2}\cdot k_{1}f+f_{2}r_{2}^{4}\cdot k_{2}f=r_{2}\\ f_{3}f+f_{3}r_{3}^{2}\cdot k_{1}f+f_{3}r_{3}^{4}\cdot k_{2}f=r_{3} \end{array}\right.$$

For solving linearly, *f*, *k*_1_*f*, *k*_2_*f* are seen as the unknown parameters in Equation (9) and then a system of linear equations can be obtained as

(10) $$A_{1}X_{1}=Y_{1}$$

Here,

(11) $$\begin{array}{l} A_{1}=\left[\begin{array}{ccc} f_{1} & f_{1}r_{1}^{2} & f_{1}r_{1}^{4}\\ f_{2} & f_{2}r_{2}^{2} & f_{2}r_{2}^{4}\\ f_{3} & f_{3}r_{3}^{2} & f_{3}r_{3}^{4} \end{array}\right]\\ X_{1}={\left[\begin{array}{ccc} f & k_{1}f & k_{2}f \end{array}\right]}^{T}\\ Y_{1}={\left[\begin{array}{ccc} r_{1} & r_{2} & r_{3} \end{array}\right]}^{T} \end{array}$$

The system can be solved linearly using $X_{1}=A_{1}^{-1}Y_{1}$. Then the focal length and the radial distortion coefficients are given respectively as follows:

(12) $$\begin{array}{l} f=X_{1}\left(1\right)\\ k_{1}=\frac{X_{1}\left(2\right)}{X_{1}\left(1\right)}\\ k_{2}=\frac{X_{1}\left(3\right)}{X_{1}\left(1\right)} \end{array}$$

(2) The traditional model. This two-parameter model is given by the formula

(13) $$p_{i}=p_{i}^{d}\left(1+k_{1}r_{i}^{2}+k_{2}r_{i}^{4}\right)$$

Similarly, a system of linear equations can be obtained as

(14) $$A_{2}X_{2}=Y_{2}$$

Here

(15) $$\begin{array}{l} A_{2}=\left[\begin{array}{ccc} f_{1} & -r_{1}^{3} & -r_{1}^{5}\\ f_{2} & -r_{2}^{3} & -r_{2}^{5}\\ f_{3} & -r_{3}^{3} & -r_{3}^{5} \end{array}\right]\\ X_{2}={\left[\begin{array}{ccc} f & k_{1} & k_{2} \end{array}\right]}^{T}\\ Y_{2}={\left[\begin{array}{ccc} r_{1} & r_{2} & r_{3} \end{array}\right]}^{T} \end{array}$$

Then, the focal length and the radial distortion coefficients are computed linearly, as follows:

(16) $$X_{2}=A_{2}^{-1}Y_{2}$$

In this section, when the LM algorithm is used to solve the intermediate variable *f_i_*, our method does not involve the radial distortion coefficients. This means that no matter what radial distortion model is used, the focal length and distortion coefficients can be solved linearly after obtaining the intermediate variable *f_i_*.

### 2.3. Camera Pose Estimation

**Figure 3:**
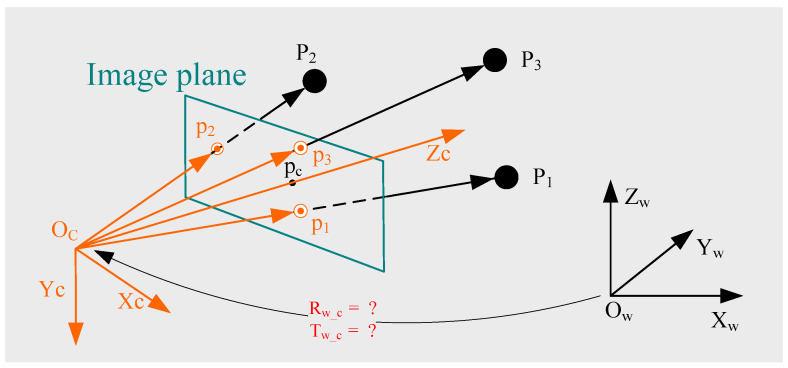
Camera position estimation from the valid 2D–3D point correspondences and camera position.

The positions of undistorted points can be obtained according to the radial distortion coefficients estimated in Section 2.2, and the positions of distorted points in the original image. Then, the valid 2D–3D point correspondences can be obtained, and these are illustrated in Figure 3.

In Figure 3, *O_c_*-*X_c_Y_c_Z_c_* is the original camera frame and *O_w_*-*X_w_Y_w_Z_w_* is the original world frame. Two 2D–3D point correspondences can be used to estimate camera pose with a known camera position, focal length and radial distortion, and then a single solution can be obtained [37]. Alternatively, three 2D–3D point correspondences can be used to estimate camera poses with a known focal length and radial distortion, and up to four solutions can be obtained [15]. In this paper, to obtain the single solution directly, we use two 2D–3D point correspondences to estimate camera pose, i.e., rotation matrix *R_w_c_* and translation vector *T_w_c_*, written in red in Figure 3.

Here, we define a new camera frame *O_c_*-*X_c_*_2_*Y_c_*_2_*Z_c_*_2_ and a new world frame *O_c_*-*X_w_*_2_*Y_w_*_2_*Z_w_*_2_. The new camera frame is defined as follows:

(17) $$\begin{array}{l} \vec{O_{c}X_{c2}}=\frac{\vec{O_{c}p_{1}}}{\left\|\vec{O_{c}p_{1}}\right\|}\\ \vec{O_{c}Z_{c2}}=\frac{\vec{O_{c}X_{c2}}\times \vec{O_{c}p_{2}}}{\left\|\vec{O_{c}X_{c2}}\times \vec{O_{c}p_{2}}\right\|}\\ \vec{O_{c}Y_{c2}}=\vec{O_{c}Z_{c2}}\times \vec{O_{c}X_{c2}} \end{array}$$

Here, $\vec{O_{c}p_{i}}=\left[\begin{array}{ccc} u_{i} & v_{i} & f \end{array}\right]$, which can be obtained after the radial distortion and focal length have been estimated in Section 2.2. In the new camera frame, the *X_c_*_2_ axis is the vector $\vec{O_{c}p_{1}}$, the *Z_c_*_2_ axis is perpendicular to the plane *O_c_p*_1_*p*_2_, and the *Y_c_*_2_ axis is defined by the right-handed coordinate system. Then, the point *Pc* in the original camera frame *O_c_*-*X_c_Y_c_Z_c_* can be transformed to point *P_c_*_2_ in the new world frame *O_c_*-*X_c_*_2_*Y_c_*_2_*Z_c_*_2_ using

(18) $$\begin{array}{l} P_{c2}=N_{c2}\cdot P_{c}\\ N_{c2}={\left[\begin{array}{ccc} \vec{O_{c}X_{c2}} & \vec{O_{c}Y_{c2}} & \vec{O_{c}Z_{c2}} \end{array}\right]}^{T} \end{array}$$

The new world frame is defined as follows:

(19) $$\begin{array}{l} \vec{O_{c}X_{w2}}=\frac{\vec{O_{c}P_{1}}}{\left\|\vec{O_{c}P_{1}}\right\|}\\ \vec{O_{c}Z_{w2}}=\frac{\vec{O_{c}X_{w2}}\times \vec{O_{c}P_{2}}}{\left\|\vec{O_{c}X_{w2}}\times \vec{O_{c}P_{2}}\right\|}\\ \vec{O_{c}Y_{w2}}=\vec{O_{c}Z_{w2}}\times \vec{O_{c}X_{w2}} \end{array}$$

In the new world frame, the origin is the camera position *O_c_*, which is known, the *X_w_*_2_ axis is the vector $\vec{O_{c}P_{1}}$, the *Z_w_*_2_ axis is perpendicular to the plane *O_c_P*_1_*P*_2_ and the *Y_w_*_2_ axis is defined by right-handed coordinate system. Then the point *P_w_* in the original world frame *O_w_*-*X_w_Y_w_Z_w_* can be transformed to point *P_w_*_2_ in the new world frame *O_c_*-*X_w_*_2_*Y_w_*_2_*Z_w_*_2_ using

(20) $$\begin{array}{l} P_{w2}=N_{w2}\cdot \left(P_{w}-O_{c}\right)\\ N_{w2}={\left[\begin{array}{ccc} \vec{O_{c}X_{w2}} & \vec{O_{c}Y_{w2}} & \vec{O_{c}Z_{w2}} \end{array}\right]}^{T} \end{array}$$

**Figure 4:**
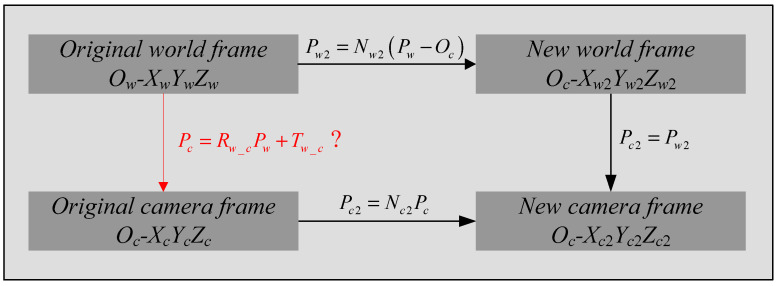
The transformations between each pair of frames. The transformation written in red is what needs to be solved in this section.

Obviously, the new camera frame and the new world frame coincide. Then, we assume that point *P_c_* in the original camera frame and point *P_w_* in the original world frame are the same point, and according to the definitions of the new camera frame and new world frame, we can obtain the transformations between each two frames, as shown in Figure 4.

Then, the rotation matrix *R_w_c_* and translation vector *T_w_c_* can be obtained from Figure 4, as follows:

(21) $$\begin{array}{l} R_{w\_c}=N_{c2}^{-1}\cdot N_{w2}\\ T_{w\_c}=-N_{c2}^{-1}\cdot N_{w2}\cdot O_{c} \end{array}$$

Pose estimation is thus finished.

## 3. Experiments and Results

In this Section, the numerical stability, noise sensitivity, computational speed and robustness to camera position noise of our proposed method, using the division model and traditional model, respectively, are thoroughly tested in the synthetic data, and compared to the general solver used in [21] (Josephson’s method). Josephson’s method is fast and numerically stable, and is the first method used to estimate camera pose with unknow focal length and radial distortion from four 2D–3D point correspondences. Then, real images are used to test the feasibility of our proposed method in real scenarios. From the experiments, we can see that the results of the division model and the traditional model are basically the same. As such, only the result of the division model is discussed in this section.

### 3.1. Synthetic Data

A virtual perspective camera with radial distortion is synthesized. Its image resolution is 1280 × 800 pixels and the center of the image is the principal point, i.e., the center of distortion in this paper. Then, the 3D points of synthetic data are randomly generated in a box of [−20, 20] × [−20, 20] × [180, 220], and the 2D image points of the synthetic data are generated by projecting the 3D points using the virtual camera. Now we can randomly generate 2D–3D point correspondences for testing the numerical stability, noise sensitivity, computational speed and robustness to camera position noise of our proposed method.

#### 3.1.1. Numerical Stability

**Figure 5:**
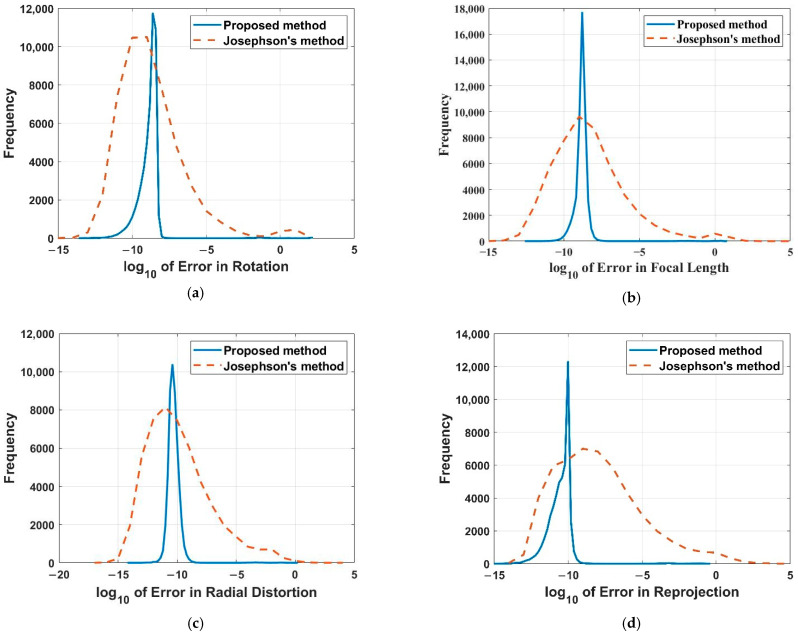
Numerical stability with errors in rotation (**a**), focal length (**b**), radial distortion (**c**) and reprojection (**d**) for our proposed method (Blue) and Josephson’s method (Yellow).

Three 2D–3D point correspondences without noise are randomly generated for our proposed method, and four are randomly generated for Josephson’s method. 50,000 trials are performed independently, and the distributions of the log10 value of error in rotation, focal length, radial distortion and reprojection are reported, as shown in Figure 5.

From Figure 5, it can be inferred that the error distribution of our proposed method is more concentrated compared to Josephson’s method. In addition, in terms of focal length, radial distortion and reprojection, our proposed method has better numerical stability than Josephson’s method. In terms of rotation, the performance of our proposed method is almost the same as Josephson’s method.

#### 3.1.2. Noise Sensitivity

**Figure 6:**
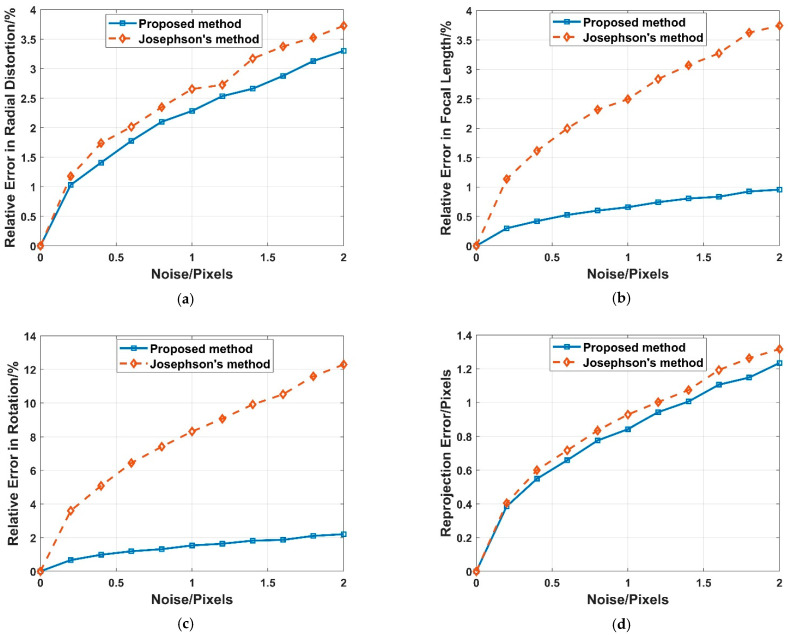
Noise sensitivity with median error in radial distortion (**a**), focal length (**b**), rotation (**c**) and reprojection (**d**) for our proposed method (Blue) and Josephson’s method (Yellow).

Three 2D–3D point correspondences with zero-mean Gaussian noise are randomly generated for our proposed method, and four are randomly generated for Josephson’s method. The noise deviation level varies from 0 to 2 pixels. Then 50,000, trials are performed independently, and the median values of error in radial distortion, focal length, rotation and reprojection are reported, as shown in Figure 6.

Obviously, as the noise increases, so do the errors of the proposed method and Josephson’s method. The proposed method has better numerical stability. In terms of the focal length and rotation, the proposed method performs much better than Josephson’s method. In terms of the radial distortion and reprojection, the proposed method performs slightly better than Josephson’s method.

#### 3.1.3. Computational Speed

We test our proposed method on a 3.3 GHz two-core laptop. Three 2D–3D point correspondences without noise are randomly generated for our proposed method and four are randomly generated for Josephson’s method. Then, 50,000 trials are performed independently, and the medians of the computational times of our proposed method and Josephson’s method are 0.0768 s and 0.0743 s, respectively. It can be seen that our proposed method is 3.4% slower than the general solver.

#### 3.1.4. Robustness to Camera Position Noise

**Figure 7:**
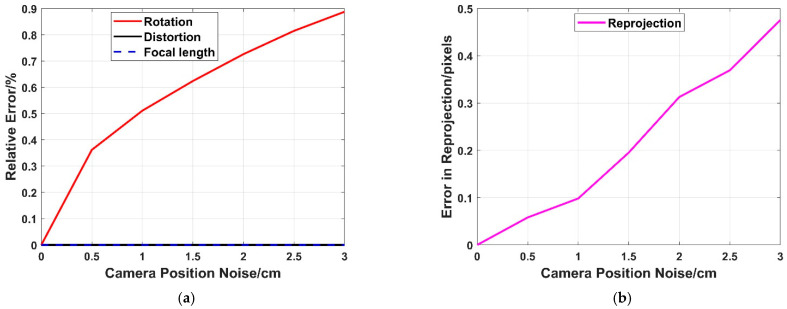
Robustness to camera position noise. (**a**) The relative error in rotation (Red), radial distortion (Black) and focal length (Blue). (**b**) The error in reprojection (purple).

With our proposed method, the difference is that it uses the camera position as prior knowledge, compared to the existing methods. The camera position is thus an important parameter, and it is necessary to analyze the effect of camera position noise on the performance of our proposed method. The camera position is generally given by RTK or the total station in this paper, and the accuracy of both these measures is better than 3 cm [56]. This section adds zero-mean Gaussian noise onto the camera’s position, whose noise deviation level varies from 0 to 3 cm. Then, 50,000 trials are performed independently, and the medians of the relative error in rotation, distortion and focal length, as well as the median of error in reprojection, are reported respectively in Figure 7.

It can be seen in Figure 7a that the relative errors in radial distortion and focal length are both close to zero. As described in Section 2, the problem is decomposed into two sub-problems, and the first sub-problem is to estimate the focal length and radial distortion. Hence, the camera position noise has almost no effect on the focal length and radial distortion estimation.

The second sub-problem is to estimate the camera pose (rotation), and we can see in Figure 7a that the relative error in rotation increases with the increase in camera position noise. This means that camera position noise has an effect on camera pose estimation. However, the maximum relative error of rotation is less than 0.9% when the camera position noise is 3 cm, which indicates that we can still obtain good results for camera pose even if there is camera position noise.

Furthermore, the relative error in rotation, radial distortion and focal length will further affect the error in reprojection. It can be seen in Figure 7b that the error in reprojection increases with the increase in camera position noise. From the previous analysis, we can see that this is mainly caused by the error in rotation. Although the camera position noise has an effect on the reprojection, the maximum error is less than 0.5 pixels, which indicates our proposed method still performs well, even though the camera position noise is present.

### 3.2. Real Images

**Figure 8:**
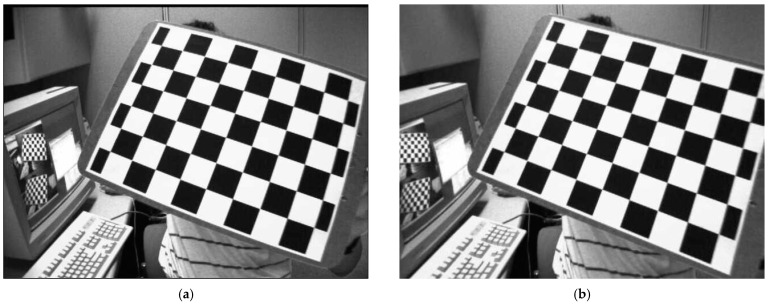
The distorted image (**a**) and undistorted image (**b**) using our method.

The preceding section tested our proposed method on synthetic data, and this section will test our proposed method on real images. Two approaches are employed to show the performance of our proposed method. First, we use an image from the internet [57] that is widely used for camera calibration. This image has heavy distortion, as shown in Figure 8.

In this image, a checkerboard is inserted that has many straight lines, which will be bent under heavy distortion, as shown in Figure 8a. Then, three corners of the checkerboard are selected to estimate the camera pose, focal length and radial distortion with our proposed method. According to the results of our proposed method, we can obtain undistorted images as shown in Figure 8b. Intuitively, it can be seen that these lines revert to straight lines, which indicates our proposed method achieves good performance even under heavy distortion.

**Figure 9:**
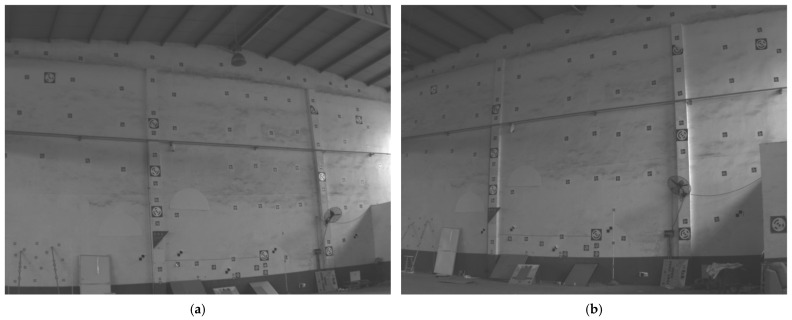
Real images with heavy distortion. (**a**) The real image captured by the first camera. (**b**) The real image captured by the second camera. Some control points are placed near the edges of both images.

The first approach shows the performance intuitively, rather than quantitatively. Hence, another approach is employed to test our proposed method with quantitative evaluation on real images. The real images are captured by two cameras (MV-CS016, the CMOS is IMX296 of Sony) with a wide-angle lens (LM6JC, the focal length is 6 mm), which gives the real images heavy distortion. Given that the further a point is from the center of the image, the heavier the distortion will be, some control points are placed near the edges of both images, and hence their projections have heavy distortion. The case is useful for testing the performance of our proposed method on radial distortion. The images are illustrated in Figure 9.

Three 2D–3D point correspondences are selected to estimate the camera pose, focal length and radial distortion by our proposed method. In this way we can obtain the undistorted images and valid 2D–3D point correspondences. In real scenarios, the ground truths of the camera pose, focal length and radial distortion are all unknown. Hence, we cannot test our proposed method directly on real images. However, the ground truths of the 3D control points given by a total station (NTS-330R, measuring precision better than 0.5 cm) are known. Hence, we can use the information to test our proposed method indirectly on real images. The measured values of these control points can be obtained by binocular vision, the accuracy of which is determined by the camera pose, focal length and radial distortion estimated via our proposed method. Binocular vision uses two cameras to obtain a three-dimensional coordinate, and this is the common three-dimensional measurement method. It can solve a problem wherein one camera cannot obtain depth information [58]. Accordingly, the accuracy of the measured values can reflect the performance of our proposed method.

We compute all the control point positions using binocular vision with our proposed method and Josephson’s method to obtain the errors between the measured values and the ground truths. The mean relative errors of our proposed method and Josephson’s method are 0.27% and 0.34%, respectively, which indicate that our proposed method achieves a better performance on real images. In addition, the mean relative error of our proposed method is very low, and this indicates that our proposed method can obtain good results on real images.

Similarly, we can obtain the mean reprojection error of the control points. The reprojection error is affected by the estimation of focal length, radial distortion and absolute pose, hence it can also reflect the performance of our proposed method. The mean reprojection with our proposed method is 0.21 pixels, and it is 0.29 pixels with Josephson’s method, which indicates our proposed method achieves better performance. This is consistent with the results derived from synthetic data, and shows our proposed method performs well on both synthetic data and real images.

## 4. Discussion

This paper proposes a new method for absolute camera pose estimation when the focal length and radial distortion are unknown, from only three 2D–3D point correspondences and a known camera position. Up to four-order radial distortion can be estimated. The proposed method is especially suitable for cases wherein wide-angle and zoom lenses are used. The differences and advantages of the proposed method will be discussed in the following section.

### 4.1. Difference and Advantage

Estimating camera pose or some intrinsic parameters (i.e., focal length and radial distortion in this paper) from 2D–3D point correspondences is an important step in computer vision. For absolute pose estimation, the position of a 3D point in the world frame must be known first, which is difficult in practical applications. Hence, using fewer 2D–3D point correspondences is the aim of researchers, and is also why we are undertaking the work in this paper. Although it is difficult to obtain the absolute position of a 3D point in a world frame, it is easier to obtain the absolute position of a camera using positioning devices (e.g., IMU, RTK and total station). This is also the reason why our proposed method can use fewer 2D–3D point correspondences compared to traditional methods. Most of the traditional methods used for determining the camera pose, focal length and distortion estimation are based on the projection matrix, which is used to obtain a system of polynomial equations, and estimate the unknown parameters directly. In addition, most methods use the division distortion model to simplify the system. The difference in this paper is that the system of polynomial equations is obtained from the geometry of the photogrammetry, not the projection transformation, as in the traditional methods used for radial distortion estimation. An important geometric characteristic of radial distortion is that the orientation of the image point with respect to the center of distortion (i.e., the principal point in this paper) under radial distortion is unchanged, and this is then used to obtain a system of polynomial equations, which is the most interesting point of our proposed method. Lastly, the LM algorithm is used to solve the intermediate variables, and does not solve the radial distortion directly. This means that no matter what radial distortion model is used, the focal length and distortion coefficients can be solved linearly after obtaining the intermediate variables. It can be seen that the major difference between the proposed method and the traditional methods is that the former starts with geometry, and the latter starts with projection transformation.

Since values with no radial distortion are used as the initial solutions, our proposed method returns only one solution, but up to four are returned by Josephson’s method, and it thus needs an extra constraint to disambiguate the multi-solution phenomena. In addition, the initial solutions of the LM algorithm in our proposed method are the values of the non-distorted model. Since the initial solutions are close to the truth values, they are highly likely to converge on the global optimal solution. Then, we carry out a simulation and an experiment in Section 3, and the results show the feasibility of our proposed method.

Our proposed method uses some known extrinsic parameters, i.e., camera position in this paper, which means the camera’s position is an essential factor for numerical stability and noise sensitivity. The camera position is given with high precision by RTK or total station, and hence has a low error of 0–3 cm. As described in Section 3.1.4, our proposed method has good robustness to camera position noise. The good robustness is the reason why our proposed method achieves better performance in terms of numerical stability and noise sensitivity. Furthermore, a good initial solution is utilized, and this can be given by using the general PnP solvers without distortion, which cannot access the globally optimal solution. In this paper, the good initial solution is the main reason why our method has better numerical stability and noise sensitivity. Last, the solving process mainly involves a linear solution, except for the intermediate variable. This is another reason why our proposed method has lower error, as described in Section 3.1.2.

Since our proposed method achieves better performance in terms of numerical stability and noise sensitivity, and the camera position is given with high precision by a total station, we can obtain good results in the measurement of point position and reprojection for real images.

However, the major drawback of our proposed method is that it is 3.4% slower than the general solver. This drawback is caused by the low iteration step size. If we increase the step size to increase the computational speed and make our proposed method come close to the general solver in accuracy, our method will be 17.6% faster than the general solver. It can be seen that the reason our proposed method is slightly slower, as described in Section 3.1.3, is that this improves its accuracy. In practical applications, depending on the need for accuracy, we can change the step size of the iterations to increase or decrease the computational speed.

### 4.2. Future Work

In this paper, the iteration step size of the LM algorithm makes a profound impact on the computational speed and accuracy. Currently, our proposed method chooses the step size based on experimental experience. Hence, the work that we will do in the future will establish a method for adapting the iteration step size, which will choose the most appropriate step size automatically in order to balance the relationship between computational speed and accuracy.

## 5. Conclusions

We have proposed a new method to estimate the camera pose, focal length and radial distortion simultaneously using three 2D–3D point correspondences. This method has two key features that enable it to obtain a single solution efficiently and accurately. The first key feature is that the important geometric characteristic of radial distortion, which is the orientation of the image point with respect to the center of distortion under radial distortion, is unchanged, and this is used to solve our problem. Then, the focal length and up to four-order radial distortions can be determined iteratively with this geometric characteristic, and applied to multiple distortion models. The second feature is that the values with no radial distortion are the initial values, which are close to the global optimal solutions. This means that our problem can be efficiently and accurately solved with the initial values.

The experimental results indicate that our proposed method performs well in terms of numerical stability and noise sensitivity for synthetic and real data. It is particularly suitable for cases wherein a wide-angle or zoom lens with heavy distortion is used. 

## Data Availability

The data presented in this study are available in the manuscript.

## References

[1] Kukelova Z., Heller J., Bujnak M., FitzGibbon A., Pajdla T. Efficient solution to the epipolar geometry for radially distorted cameras. Proceedings of the IEEE International Conference on Computer Vision.

[2] Sweeney C.M. (2016). Modeling and Calibrating the Distributed Camera.

[3] Camposeco F., Sattler T., Pollefeys M. Non-parametric structure-based calibration of radially symmetric cameras. Proceedings of the IEEE International Conference on Computer Vision.

[4] Kukelova Z., Heller J., Bujnak M., Pajdla T. Radial distortion homography. Proceedings of the IEEE Conference on Computer Vision and Pattern Recognition.

[5] Larsson V., Sattler T., Kukelova Z., Pollefeys M. Revisiting radial distortion absolute pose. Proceedings of the IEEE International Conference on Computer Vision.

[6] Jiang F., Kuang Y., Solem J.E., Åström K. A minimal solution to relative pose with unknown focal length and radial distortion. Proceedings of the Asian Conference on Computer Vision.

[7] Nakano G. A versatile approach for solving PnP, PnPf, and PnPfr problems. Proceedings of the European Conference on Computer Vision.

[8] Wu Y., Tang F., Li H. (2018). Image-based camera localization: An overview. Vis. Comput. Ind. Biomed. Art.

[9] Sattler T., Sweeney C., Pollefeys M. On sampling focal length values to solve the absolute pose problem. Proceedings of the European Conference on Computer Vision.

[10] Zheng Y., Kuang Y., Sugimoto S., Astrom K., Okutomi M. Revisiting the pnp problem: A fast, general and optimal solution. Proceedings of the IEEE International Conference on Computer Vision.

[11] Ferraz L., Binefa X., Moreno-Noguer F. Very fast solution to the PnP problem with algebraic outlier rejection. Proceedings of the IEEE Conference on Computer Vision and Pattern Recognition.

[12] Bujnák M. (2012). Algebraic Solutions to Absolute Pose Problems. Ph.D. Thesis.

[13] Youyang F., Qing W., Yuan Y., Chao Y. (2019). Robust improvement solution to perspective-n-point problem. Int. J. Adv. Robot. Syst..

[14] Wolfe W., Mathis D., Sklair C., Magee M. (1991). The perspective view of three points. IEEE Trans. Pattern Anal. Mach. Intell..

[15] Wang P., Xu G., Wang Z., Cheng Y. (2018). An efficient solution to the perspective-three-point pose problem. Comput. Vis. Image Underst..

[16] Gao X.S., Hou X.R., Tang J., Cheng H.F. (2003). Complete solution classification for the perspective-three-point problem. IEEE Trans. Pattern Anal. Mach. Intell..

[17] Triggs B. Camera pose and calibration from 4 or 5 known 3d points. Proceedings of the Seventh IEEE International Conference on Computer Vision.

[18] Larsson V., Kukelova Z., Zheng Y. Making minimal solvers for absolute pose estimation compact and robust. Proceedings of the IEEE International Conference on Computer Vision.

[19] Kukelova Z., Albl C., Sugimoto A., Schindler K., Pajdla T. Minimal rolling shutter absolute pose with unknown focal length and radial distortion. Proceedings of the European Conference on Computer Vision.

[20] Bujnak M., Kukelova Z., Pajdla T. New efficient solution to the absolute pose problem for camera with unknown focal length and radial distortion. Proceedings of the Asian Conference on Computer Vision.

[21] Josephson K., Byrod M. Pose estimation with radial distortion and unknown focal length. Proceedings of the IEEE Conference on Computer Vision and Pattern Recognition.

[22] Huang K., Ziauddin S., Zand M., Greenspan M. One shot radial distortion correction by direct linear transformation. Proceedings of the IEEE International Conference on Image Processing.

[23] D’Alfonso L., Garone E., Muraca P., Pugliese P. On the use of IMUs in the PnP Problem. Proceedings of the International Conference on Robotics and Automation.

[24] Ornhag M.V., Persson P., Wadenback M., Astrom K., Heyden A. Efficient real-time radial distortion correction for UAVs. Proceedings of the IEEE/CVF Winter Conference on Applications of Computer Vision.

[25] Kukelova Z., Bujnak M., Pajdla T. Closed-form solutions to minimal absolute pose problems with known vertical direction. Proceedings of the Asian Conference on Computer Vision.

[26] Sweeney C., Flynn J., Nuernberger B., Turk M., Höllerer T. Efficient computation of absolute pose for gravity-aware augmented reality. Proceedings of the IEEE International Symposium on Mixed and Augmented Reality.

[27] Chang Y.J., Chen T. Multi-view 3D reconstruction for scenes under the refractive plane with known vertical direction. Proceedings of the International Conference on Computer Vision.

[28] D’Alfonso L., Garone E., Muraca P., Pugliese P. P3P and P2P problems with known camera and object vertical directions. Proceedings of the Mediterranean Conference on Control and Automation.

[29] Kukelova Z., Pajdla T. A minimal solution to the autocalibration of radial distortion. Proceedings of the IEEE Conference on Computer Vision and Pattern Recognition.

[30] Oskarsson M. Fast solvers for minimal radial distortion relative pose problems. Proceedings of the IEEE/CVF Conference on Computer Vision and Pattern Recognition.

[31] Barreto J.P., Daniilidis K. Fundamental matrix for cameras with radial distortion. Proceedings of the Tenth IEEE International Conference on Computer Vision.

[32] Kuang Y., Solem J.E., Kahl F., Astrom K. Minimal solvers for relative pose with a single unknown radial distortion. Proceedings of the IEEE Conference on Computer Vision and Pattern Recognition.

[33] Steele R.M., Jaynes C. Overconstrained linear estimation of radial distortion and multi-view geometry. Proceedings of the European Conference on Computer Vision.

[34] Fitzgibbon A.W. Simultaneous linear estimation of multiple view geometry and lens distortion. Proceedings of the 2001 IEEE Computer Society Conference on Computer Vision and Pattern Recognition.

[35] Kukelova Z., Bujnak M., Pajdla T. Real-time solution to the absolute pose problem with unknown radial distortion and focal length. Proceedings of the IEEE International Conference on Computer Vision.

[36] Guo K., Ye H., Gu J., Chen H. (2021). A novel method for intrinsic and extrinsic parameters estimation by solving perspective-three-point problem with known camera position. Appl. Sci..

[37] Guo K., Ye H., Zhao Z., Gu J. (2021). An efficient closed form solution to the absolute orientation problem for camera with unknown focal length. Sensors.

[38] Sturm P., Ramalingam S. (2011). Camera Models and Fundamental Concepts Used in Geometric Computer Vision.

[39] Kileel J., Kukelova Z., Pajdla T., Sturmfels B. (2018). Distortion varieties. Found. Comput. Math..

[40] Kannala J., Brandt S.S. (2006). A generic camera model and calibration method for conventional, wide-angle, and fish-eye lenses. IEEE Trans. Pattern Anal. Mach. Intell..

[41] Ma L., Chen Y.Q., Moore K.L. (2003). A new analytical radial distortion model for camera calibration. arXiv.

[42] Henrique Brito J., Angst R., Koser K., Pollefeys M. Radial distortion self-calibration. Proceedings of the IEEE Conference on Computer Vision and Pattern Recognition.

[43] Wang J., Shi F., Zhang J., Liu Y. (2008). A new calibration model of camera lens distortion. Pattern Recognit..

[44] Duane C.B. (1971). Close-range camera calibration. Photogramm. Eng..

[45] Zhang Z. (2000). A flexible new technique for camera calibration. IEEE Trans. Pattern Anal. Mach. Intell..

[46] Papadaki A.I., Georgopoulos A. Development, comparison, and evaluation of software for radial distortion elimination. Proceedings of the Videometrics, Range Imaging, and Applications XIII.

[47] Remondino F., Fraser C. (2006). Digital camera calibration methods: Considerations and comparisons. Int. Arch. Photogramm. Remote Sens. Spat. Inf. Sci..

[48] Lopez M., Mari R., Gargallo P., Kuang Y., Gonzalez-Jimenez J., Haro G. Deep single image camera calibration with radial distortion. Proceedings of the IEEE/CVF Conference on Computer Vision and Pattern Recognition.

[49] Bukhari F., Dailey M.N. (2013). Automatic radial distortion estimation from a single image. J. Math. Imaging Vis..

[50] Wu F., Wei H., Wang X. (2017). Correction of image radial distortion based on division model. Opt. Eng..

[51] Byrod M., Kukelova Z., Josephson K., Pajdla T., Astrom K. Fast and robust numerical solutions to minimal problems for cameras with radial distortion. Proceedings of the IEEE Conference on Computer Vision and Pattern Recognition.

[52] Kukelova Z., Pajdla T. Two minimal problems for cameras with radial distortion. Proceedings of the IEEE 11th International Conference on Computer Vision.

[53] Wang A., Qiu T., Shao L. (2009). A simple method of radial distortion correction with center of distortion estimation. J. Math. Imaging Vis..

[54] Wang Q., Wang Z.Y., Smith T. (2016). Radial distortion correction in a vision system. Appl. Opt..

[55] Kim J., Bae H., Lee S.G. (2021). Image distortion and rectification calibration algorithms and validation technique for a stereo camera. Electronics.

[56] Forlani G., Dall’Asta E., Diotri F., di Cella U.M., Roncella R., Santise M. (2018). Quality assessment of DSMs produced from UAV flights georeferenced with on-board RTK positioning. Remote Sens..

[57] Camera Calibration Toolbox. http://www.vision.caltech.edu/bouguetj/calib_doc/htmls/example5.html.

[58] Do P.N.B., Nguyen Q.C. A review of stereo-photogrammetry method for 3-D reconstruction in computer vision. Proceedings of the IEEE 19th International Symposium on Communications and Information Technologies.

